# Targeting *KRAS* in metastatic colorectal cancer: current strategies and emerging opportunities

**DOI:** 10.1186/s13046-018-0719-1

**Published:** 2018-03-13

**Authors:** Manuela Porru, Luca Pompili, Carla Caruso, Annamaria Biroccio, Carlo Leonetti

**Affiliations:** 1 UOSD SAFU, Regina Elena National Cancer Institute, Rome, Italy; 2 University of Tuscia, Viterbo, Italy; 3 Oncogenomic and Epigenetic Unit, Regina Elena National Cancer Institute, Rome, Italy

## Abstract

Developing drugs that target *KRAS*, the most frequently mutated oncogene in cancer, has not been successful despite much concerted efforts dedicated towards it in the last thirty years. Considering the key role this driver oncogene plays, the pharmacological drugging of *KRAS* remains a key challenge for cancer research. In this review, we highlight the emerging experimental strategies for blocking *KRAS* function and signaling and its direct targeting. We also report on the results in this field of research produced by our group.

## Introduction

Colorectal cancer (CRC) is one of the most common cancers worldwide with 1.4 million new cases and 694.000 deaths reported in 2012 and where it is the fourth most deadly cancer after lung, liver and stomach [1]. Interestingly, the last decade has seen a decrease in the number of new CRC cases particularly in Western countries due to a variety of reasons including the implementation of colonoscopies as screening tests, the use of chemopreventive agents such as non-steroidal anti-inflammatory drugs (NSAIDs) and the clustering of diets, nutritional supplements and regular exercise in a healthy lifestyle [2–4].

At the same time, the outcome of CRC patients, in particular that of metastatic CRC (mCRC) patients, has markedly improved given that the median overall survival more than doubled in the last twenty years reaching approximately 30 months. This amelioration in the treatment of mCRC has been obtained by combining different cytotoxic drugs, in particular the protracted infusion of 5-fluorouracil modulated by leucovorin in combination with irinotecan (FOLFIRI) or with oxaliplatin (FOLFOX), capecitabine and oxaliplatin combination (XELOX) or 5-fluorouracil, leucovorin, irinotecan and oxaliplatin (FOLFOXIRI) [5, 6] and subsequently, by the introduction of targeted-therapy [7].

As for other types of cancer, genomic instability plays a major role in CRC and where three different groups have been identified as the pathogenic mechanisms, including mainly microsatellite instability (MSI), CpG island methylator phenotype (CIMP) and chromosomal instability (CIN), which represents up to 80–85% of the causes of all CRC cases [8]. Within these types, affected pathways have been reported to be involved in cell proliferation and survival such as WNT, MAPK/PI3K, TGF-β, TP53 and mutations in different genes including *c-MYC*, *BRAF*, *PIK3CA*, *PTEN*, *SMAD2* and *SMAD4* and finally *RAS*.

**Fig. 1:**
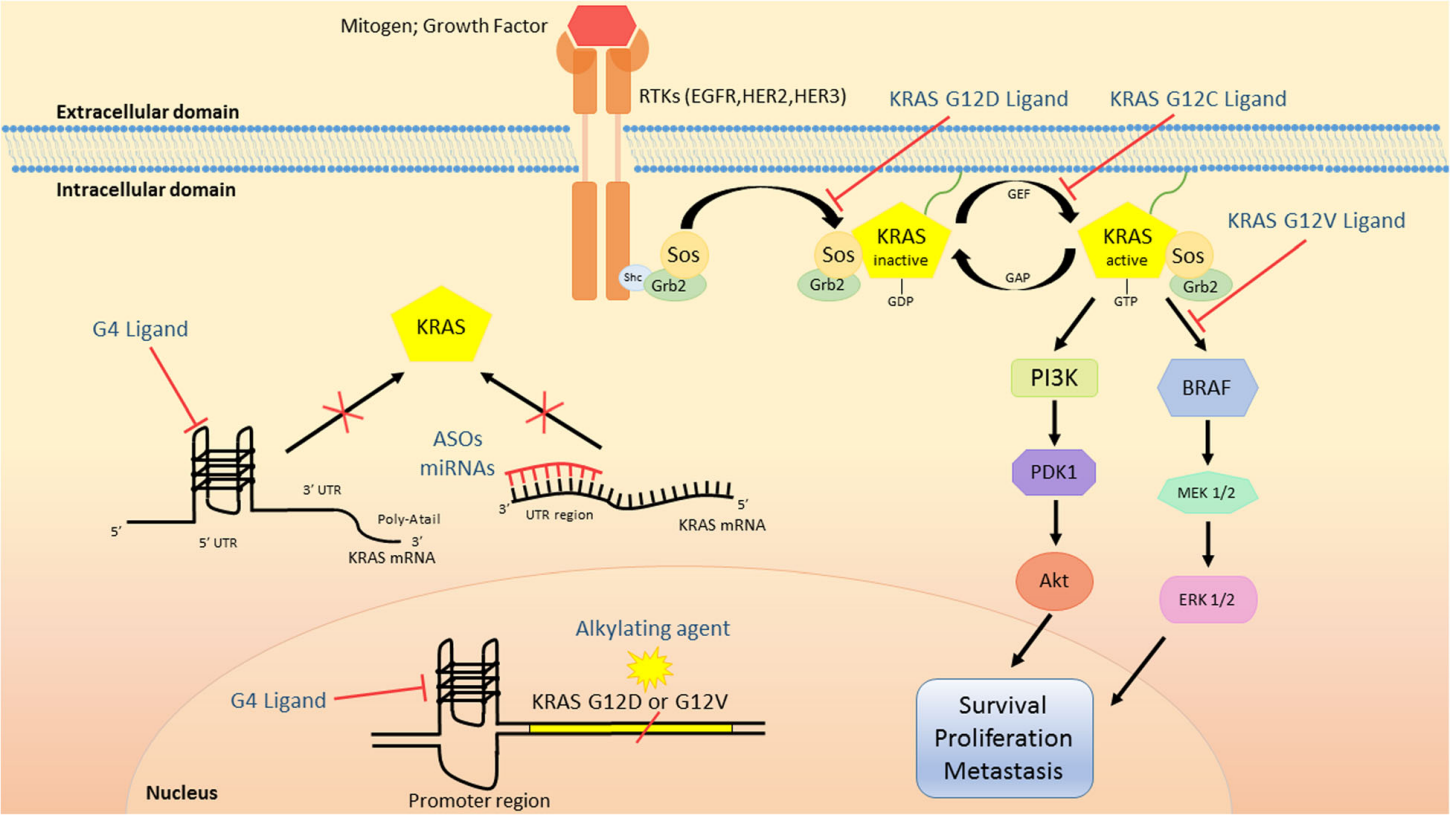
Direct targeting of KRAS. ASOs: Antisense Oligonucleotides; GAP: GTP-ase Activating Proteins; GEF: Guanine Nucleotide Exchange Factor; G4: G-quadruplex; RTKs: Receptor Tyrosine Kinases

The three human *RAS* genes (*KRAS*, *NRAS* and *HRAS*) are the most frequently mutated oncogenes in human cancer appearing in 90% of pancreatic, 35% of lung and in 45% of colon cancers. These high occurrences make *RAS* one of the most important targets in oncology for drug development [9]. In particular, KRAS is the isoform prevalently mutated in pancreas, lung and colon cancer, while NRAS is the predominant isoform mutated in cutaneous melanomas and acute myelogenous leukemia and HRAS is the predominant isoform mutated in the bladder [9]. The reasons for this high prevalence of *RAS* mutation in cancers and for the preferential mutation in some kind of cancers still remains to be elucidated. The three human *RAS* genes that encode four small guanosine triphosphatase (GTPases) are KRAS4A, KRAS4B, HRAS and NRAS. RAS is the component of the mitogen activated protein kinase (MAPK) signaling pathway, which is activated by a ligand binding to a receptor tyrosine kinase (RTK) such as the epidermal growth factor receptor (EGFR). RAS exists in the non-active (GDP, guanosine diphosphatase) or active-state (GTP) and the transition between these two states is responsible for signal transduction events occurring from the cell surface receptor to the inside of the cell which is crucial for cell growth and differentiation [9]. In physiological conditions, this switch is catalysed by two guanine exchange factors known as SOS1/2, that promote the activation of RAS proteins by stimulating GDP for GTP exchange and GTPase-activating proteins (GAPs), which in turn accelerate RAS-mediated GTP hydrolysis. The GTP-bound form of RAS is the activated state and, together with the insensitivity to cytosolic GAP [9], represents the biochemical key defect of mutant RAS proteins, which results in persistent accumulation of the active, GTP-bound protein and activation of multiple downstream effectors (Fig. 1).

Mutated *KRAS* is a major driver for malignant transformation in pancreatic tumors and in lung adenocarcinoma, as G12C mutations are detected in early lesions, retained in all metastases and are a hallmark in the exposure to tobacco smoke, respectively [10]. Regarding CRC, although *KRAS* mutations occur as an early event in about 50% of cases, they are probably not the primary initiating events being but the loss of APC or mutations in β-catenin in mismatch repair deficient tumors. The degree to which these tumors depend on *KRAS* is still underinvestigation [11] but these high occurrences make *RAS* one of the most important drug targets for cancer, including CRC.

### Emerging biomarkers of resistance to anti-EGFR treatment

Most human epithelial cancer are characterized by the functional activation of growth factors and receptors of the epidermal growth factor receptor (EGFR) family. EGFR is a receptor tyrosine kinase which, by triggering a series of intracellular signals, controls proliferation of cancer cells, differentiation, angiogenesis and metastasis dissemination. In particular, 60–80% of patients with CRC overexpress EGFR, this has been associated with the progression of the malignancy and with poor prognosis. A clinical benefit for mCRC patients has been observed after the approval of humanized monoclonal antibodies cetuximab and panitumumab, directed against the extracellular domain of the EGFR, which block ligand binding and lead to inhibition of the downstream RAS-RAF-MEK-ERK signaling pathway. Subsequently, it was observed that EGFR status did not predict the efficacy of treatment [7]. In contrast, it has been found that resistance to anti-EGFR therapy is mediated by mutations in *KRAS* gene which result in constitutive activation of the RAS-RAF-MEK-ERK pathway. This means that *RAS* testing is mandatory before treatment with anti-EGFR therapy and and *RAS* analysis should include at least *KRAS* exons 2, 3 and 4 (codons 12, 13, 59, 61, 117 and 146) and *NRAS* exons 2, 3 and 4 (codons 12, 13, 59, 61 and 117) [7]. In addition, the presence of *BRAF* V600E mutation, which is found in 8–12% of patients with mCRC, is almost exclusively non-overlapping with *RAS* mutations and should be assessed before the initiation of therapy with anti-EGFR. In fact, there are accumulating data showing that *BRAF* mutations are predictive of resistance to anti-EGFR therapy [12].

Despite wild-type *RAS,* mCRC patients initially respond to cetuximab or panitumumab, treatment is effective in less than 10% of patients and the majority of them will exhibit disease progression [13, 14], thus suggesting the onset of acquired resistance to anti-EGFR therapy. In the last-five years many biomarkers and pathways involved in anti-EGFR resistance have been identified and are continuously on the rise, mainly due to the use of novel technologies and advanced approaches.

From this perspective, the integration of the classical tumour biopsy with liquid biopsy and the use of next-generation sequencing (NGS) covering hundred of genes, represents a very potent approach that permits to dissect molecular tumour heterogeneity and to detect emerging mutations responsible for the failure of anti-EGFR therapy that are not captured by biopsy in single lesions. In particular, the analysis of plasma DNA permitted to Bardelli’s group [15] to reveal a novel mechanism of acquired resistance to anti-EGFR therapy in patient which initially responded and subsequently progressed. In fact, they observed the presence of *KRAS* mutated alleles in blood samples obtained at progression accompanied by *EGFR* ectodomain mutations and *KRAS* or *MET* gene amplification. Interestingly, the *KRAS* mutant alleles, which emerge at the time of disease progression, decline at the suspension of anti-EGFR therapy, thus indicating the dynamic mode of the tumor cell population that was confirmed in experimental models. Accordingly to these observations, the authors planned to re-challenge patients which progressed after anti-EGFR therapy but showed a subsequent decline of *KRAS* mutant clones in circulating tumor DNA, thus leading authors to postulate that clonal evolution of tumor cells that survive treatment with anti-EGFR antibodies, continues beyond the clinical progression. The lack of response to anti-EGFR therapy has been associated with alterations in different genes. In particular, one patient with *KRAS* and *NRAS* wild-type who initially responded to chemotherapy plus cetuximab but that successively progressed, evidenced a K57 T *MEK1* mutation in the NGS analysis of the primary tumour and liver metastasis [16]. This mutation was not observed prior to cetuximab treatment, suggesting MEK1 as a novel mechanism of acquired resistance to cetuximab. Subsequent in vitro experiments, showing that the combination with the MEK inhibitor trametinib restored sensitivity to anti-EGFR antibodies, the patient was treated with trametinib and panitumumab that elicited a reduction in size of the liver metastasis previously analyzed, however other metastatic lesions had in the meantime progressed. The analysis of circulating (ct) DNA from peripheral blood confirmed the presence of *MAP2K1* p.K57 T variants prior to starting therapy with trametinib and panitumumab and the decline of the levels in the course of treatment, thus indicating the specific targeting of *MEK1* mutant clones. Surprisingly, the ctDNA analysis revealed a *KRAS* p.Q61H mutation which lay undetected in the liver metastasis prior to therapy with trametinib and panitumumab. Moreover, the levels of mutated DNA markedly increased during treatment, thus suggesting the pre-existence of resistant *KRAS*-mutant clone responsible for the failure of cetuximab-based therapy.

Very recently, Pietrantonio et al. [17] analyzed tumor biopsies and ctDNA of 22 *RAS*-*BRAF*-wild-type, *HER2*/*MET*-negative mCRC patients, which benefited from anti-EGFR treatment and then developed resistance. Interestingly, this is the first study in which acquired resistance was studied in the tissue and the liquid biopsy of the same patient. They observed that the occurrence of anti-EGFR resistance was associated with *RAS* mutation and *HER2*/*MET* amplification and in particular an intralesion and interlesion heterogeneity of the resistance mechanism, thus highlighting the complexity of clonal evolution of resistance to EGFR-blockade. To this purpose, this high genetic heterogeneity of mCRC permits the authors to point out the differences with mutated-EGFR adenocarcinoma resistant to first generation TKIs, but sensitive to third generation of TKIs targeting T790-mutated *EGFR* [18].

All these results highlight the key role of *RAS* mutations and molecular heterogeneity of the resistance to anti-EGFR therapy and reinforce the need for more effective strategies to improve the outcome of mCRC patients.

### Approaches in targeting KRAS function and signaling

The knowledge of the biochemical defects in mutant RAS proteins was the starting point in developing the first RAS inhibitor but attempts to discover small molecules able to antagonize RAS-GTP binding failed due to the picomolar affinity of RAS for GTP and the millimolar cellular concentrations of GTP [9, 19]. These unfortunate early efforts in discovering anti-RAS drugs and the lack of well-defined hydrophobic pockets on the surface of RAS proteins, contributed to the general perception that the direct targeting of RAS may not be actionable [9]. For these reasons, indirect approaches to inhibit mutant RAS have been investigated and most advances have been represented by targeting the association of RAS with the plasma membrane, which is necessary for trafficking the protein from the cytoplasm to the inner face of the cell and for activating the effector pathway. Since the lipid modification of RAS proteins by a farnesyl isoprenoid is a key step for this association in this context, farnesyl transferase inhibitors (FTI) were developed and in particular two compounds (lonafarnib and tipifarnib) were tested in solid tumors with *KRAS* mutation in clinical trials, including CRC. Unfortunately, FTIs resulted ineffective as anti-cancer drugs in advanced phase III clinical trials, as *H-RAS*, *N-RAS* and *K-RAS* were shown to be refractory to inactivation by FTIs and can still associate with membranes as well as continue to function [20].

A recent approach towards disrupting the association of RAS with the plasma membrane is represented by the targeting of the prenyl-binding protein PDEδ which is crucial for the plasma membrane localization of prenylated RAS. Understanding the role of PDEδ in the trafficking and signaling of RAS led to the development of two small-molecules deltarasin and deltazinone, which occupy the farnesyl–binding pocket of PDEδ and inhibit the localization of Ras to plasma membrane. These compounds, used at low micromolar levels, were able to inhibit in vitro growth and tumorigenic ability of mutant *KRAS* tumor cell lines [21, 22].

The inhibition of RAF-MEK-ERK mitogen-activated kinase (MAPK) signaling pathways represents a further approach for targeting RAS activity. In this context, being RAF kinases the first to be activated in the MAPK pathway, the drug discovery led to the approval of two RAF-inhibitors in melanoma, vemurafenib and dafravenib. Paradoxically, when these compounds were evaluated in *KRAS*-mutant tumors, ERK was activated instead of being inhibited as expected. This effect was explained by the fact that vemurafenib and dabrafenib are selective for *BRAF* only, while, in *KRAS*-mutant tumors, BRAF inhibitors drive BRAF-CRAF binding which finally activated ERK [19, 23]. Based on these lines of evidence, pan-inhibitors are being developed such as LY3009120 and PLX8394, which inhibit BRAF and CRAF isoforms with similar affinity and do not activate the MAPK pathway when a *RAS* mutation is present. In particular, these compounds showed activity against in vitro cultured cells and in xenografts from tumors carrying *KRAS*, *NRAS*, or *BRAF* mutation [24–27].

### Direct targeting of KRAS

Despite several challenges and many failed attempts in targeting *KRAS*, researchers remain determined to search for new and effective approaches, and where the most promising of them will be discussed in the last sections of this review and highlighted in Fig. 1.

**a. Targeting of mutated sites**. Several studies have been performed to identify molecules able to bind the mutated sites of KRAS or inhibit the synthesis at the DNA level of the mutated protein and consequently blocking KRAS biological activity. In particular, groups from academic institutions or private companies are currently working on developing small molecules that are able to bind *KRAS* G12D mutant, compete with the KRAS-SOS interaction and prevent the formation of active KRAS-GTP [28, 29]. A different approach was represented by the use of Kobe0065-family compounds which inhibit the interaction between GTP-bound RAS and RAF and showed antitumor activity against CRC xenografts *KRAS* G12 V mutant [30]. As reported by Wilson and Tolias [31], these are promising compounds that could serve as a basis for the optimization of clinically active compounds.

A further strategy for developing small molecules like RAS-inhibitors, is the targeting of the cysteine in the mutated KRASG12C, thus impacting only the KRAS-mutated form. In particular, Ostrem et al. [32] have identified compounds that irreversibly bind to KRASG12C, favour the nucleotide GDP state over GTP, impair the binding to Raf and finally decrease viability and increase apoptosis of G12C-containing cancer cell lines. Following these results, Patricelli et al. [33] after testing several candidates for their activity against purified recombinant KRASG12C, as well as for their ability to engage KRASG12C in cells, identified ARS-853 as the most potent compound. The in vitro experiments demonstrated that the treatment of KRASG12C-mutated cells with ARS-853 inhibited the downstream signaling through both MAPK (including pMEK, pERK, and pRSK) and PI3K signaling (pAKT) pathways finally leading to a reduction in cell proliferation. In general, these studies provide convincing evidence that the targeting of inactive GDP-bound form could be a promising approach for generating novel and effective anti-RAS compounds. Obviously, in vivo experiments need to be performed before demonstrating that these compounds might be useful in the clinical setting. Moreover, given that G12C mutation is very rare in CRC while the most abundant is the G12D, the use of these compounds will be used only for a limited number of patients. Nevertheless, these results reinforce the idea of drugging a protein that has been deemed “undruggable” for longer.

Very recently Ross et al. [34] proposed a strategy against KRAS based on the use of the AZD4785, a 16-mer antisense oligonucleotides (ASO) complementary to the 3′ untranslated region (3′UTR) of KRAS mRNA sequences in a distinctly different region from the mutation codon sites, thus targeting both wild-type or mutant-KRAS. In particular, they used 2′-4′ constrained ethyl (cEt)-modified residues ASOs which showed a high stability and did not present the off-target toxicity effects which was the main issue of the first-generation phosphorotioate ASO [35]. The experiments performed in vitro on a panel of tumor cell lines of different histotypes including CRC, showed that despite AZD4785 induces a potent down-regulation of both wild-type and mutant KRAS protein, AZD4785 treatment reduces the proliferation only in mutant KRAS, while wild-type cells are not affected. Importantly, a selective inhibition of downstream MAPK and PI3K pathway signaling pathway was observed in mutant KRAS cells treated with AZD4785 and the inhibition of KRAS was not limited by the feedback reactivation of MAPK pathway, which could be a critical factor for the clinical use of these compounds. The experiments performed in vivo on xenografts and on PDXs demonstrate the high therapeutic efficacy of AZD4785 delivered systematically in mice. Finally, treatment with AZD4785 was well tolerated as only minimal changes in some clinical chemistry parameters and clinical pathology profiles were observed in mice and monkeys, thus confirming the potential use of this direct anti-KRAS strategy in the clinical setting [34].

To inhibit the mutated form of *KRAS*, Hiraoka et al. [36] proposed the use of a synthetic alkylating agent called KR12 (pyrrole-imidazole polyamide indole-seco-CBI conjugate). This small molecule was designed to recognize and alkylate adenine residues on the template strand at codon 12 (GTT and GAT), exon 2 of mutant *KRAS*. The authors demonstrated that KR12 produced a selective alkylation-mediated DNA strand cleavage at the G12D/G12 V mutations and this biochemical effect is associated with a greater reduction in the proliferation of CRC cells with G12D/G12 V mutation than that observed in G12C mutant cell, since KR12 does not recognize the latter mutation. Finally, the immunoblot analysis confirmed that the biological effects observed after KR12 treatment were related to the inhibition of KRAS, as a significant reduction in KRAS protein levels and in its GST-Raf-bound, active GTP-bound form, was observed. Interestingly, the in vivo experiments performed in immunosuppressed mice implanted with HT29 (*KRAS* WT), LS180 (heterozygous 12D/WT) or SW480 (homozygous 12 V) CRC cells, demonstrated that SW480 line was the most sensitive to the KR12 treatment since a marked inhibition of tumor growth was observed, while only a slight or no antitumor effect was observed on LS180 or HT29 models, respectively. Importantly, treatment was safe as mice remained healthy during as well as after the end of treatment, thus suggesting that KR12 could be a potential candidate for applying it in humans.

**b. Targeting of G4 structures.** G-quadruplex (G4) structures, formed in guanine-rich nucleic acid sequences, are abundant in regulatory regions of the genome, particularly in the promoters of a wide range of oncogenes, tumor suppressor genes and somatic copy number alterations related to cancer development [37, 38]. In particular, three G4 motifs were reported in the human KRAS promoter, called G4-proximal, G4-middle and G4-distal based on their proximity to the transcription start site [39]. Further studies have demonstrated that the most distal regions do not form an inducible nor stable G4 structure, whereas two more of the proximal regions do, particularly the G4-middle structure which seems to be the most critical for transcriptional silencing and for the development of targeted therapeutics [40]. Furthermore, in the *KRAS* promoter i-motifs have been identified on the complementary C-rich region which act as transcription activators. These observations led to the discovery of a benzophenanthridine alkaloid following a combined effect on the Mid-region i-motif and on the stabilization of G4, which down-regulated *KRAS* and cytotoxic activity against tumor cells [41]. Other than DNA, G4 structures were found in RNA sequences, including the 5′ untranslated region (UTRs) of KRAS mRNA [42]. Given that these sequences are important for post-transcriptional regulation of gene expression, such RNA motifs represent a potential target for G4-ligand small molecules.

A promising contribution to this field of research comes from Paulo’s group [43] which identified natural alkaloids Indoloquinolines as potential G4-ligand compounds for targeting of *KRAS* in CRC. In fact, they demonstrated the high selectivity of indolo[3,2-b]quinolines with a 7-carboxylate group and three alkylamine side chains (IQ3A) against G4 structures compared to duplex DNA and the ability on decreasing *KRAS* expression in HCT116 CRC lines. Successively, the same group showed that IQ3A compounds were able to inhibit KRAS mRNA and protein expression by the interaction with G4 structures present in the *KRAS* gene promoter and as a result, a decrease in cell proliferation and apoptosis induction was reported [44]. Interestingly, the IQ3A compounds were not selective for *KRAS* mutant cells as they are active both in wild-type and mutant CRC cells. It is important to underline that these compounds need to be evaluated in more complex experimental models than established cell lines, such xenografts or PDXs models of CRC, to establish the potential use against *KRAS* mutant CRC human tumors.

For several years our group focused on the study of novel G4 ligands as potential antineoplastic drugs and their mechanism of action [45–47]. In particular, we synthesized a new molecule, namely EMICORON, which has one piperidinyl group bound to the perylene bay area, sufficient to guarantee a good selectivity and an extended aromatic core able to increase the stacking interactions with the ending tetrad of the G4 [48]. Interestingly, we demonstrated that in addition to the ability of EMICORON to target G4 structures at telomeres, this compound is able to bind and stabilize the G-rich sequences located within the promoters of *c-MYC*, *BCL-2* and *VEGFR-2*. Importantly, following EMICORON treatment the expression of genes was affected and this led to the inhibition of proliferation, induction of apoptosis and impairment of angiogenesis both in vitro and in vivo [49].

Moreover, this compound has shown promising toxicological characteristics, as we observed that treated mice were free of adverse effects, and showed a marked antitumor efficacy against different CRC preclinical models [50], including *KRAS*-mutated patient-derived xenografts (PDXs). These models, preserving the genomic integrity and tumor heterogeneity of the human tumor of origin, can better mimic the response of humans to new anticancer therapeutics [51].

**Fig. 2:**
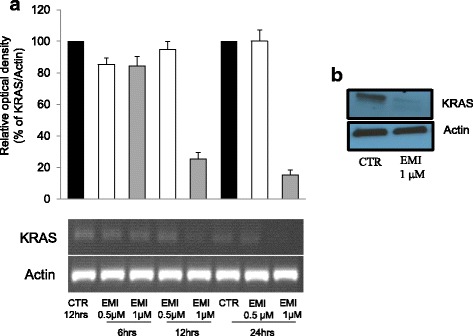
EMICORON downregulates KRAS expression. **a** HCT116 colon cancer cells were treated with EMICORON at doses of 0.5 and 1 μM for 6, 12, 24 h. The DNA extracted was amplified by PCR. The histogram shows the relative optical density of KRAS. Histograms show the mean values ± SD. A representative picture of PCR products is shown. Densitometry was performed with ImageJ software version 1.40. **b** KRAS protein levels were detected by Western blotting. The total cell extracts of HCT116 cells treated with EMICORON 1 μM for 24 h, were prepared and immunoblotting was conducted by antibodies against KRAS (Santa Cruz) or Actin (Santa Cruz)

**Fig. 3:**
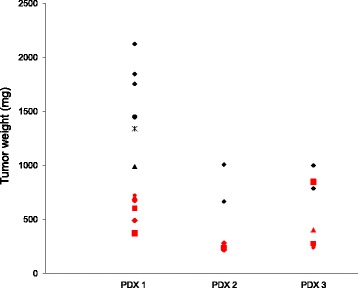
EMICORON has antitumoral activity against CRC *KRAS*-mutated PDXs**.** PDXs were obtained by the implant in mice of tumor fragments from three CRC patients at IRCCS Regina Elena National Cancer Institute (Rome, Italy). Briefly, surgical specimens, not required for histopathologic analysis, were placed in medium supplemented with antibiotics, diced into 15–20 mm^3^ pieces, coated in Matrigel and implanted in NOD.SCID mice by a small incision and subcutaneous pocket made in one side of the lower back [50]. After mass formation in mice (Passage 0), tumors were passed in four mice (Passage 1) and afterwards expanded in further mice for the drug experimentation (Passage 2). When tumors reached a mass of 250–300 mm^3^, EMICORON was administered per os at 15 mg/kg for seven days. Tumor growth was followed by caliper measurements. The tumor weight in untreated or EMICORON-treated mice was reported at the nadir of the effect. PDX 1 experiment included six untreated and six EMICORON-treated mice; PDX 2 experiment included two untreated and four EMICORON-treated mice; PDX 3 experiment included two untreated and four EMICORON-treated mice. The use of human specimens was approved by the Ethics Committee of the IRCCS Regina Elena National Cancer Institute (N. 823/2016) and animal procedures were in compliance with the national and international directives (D.L. March 4, 2014, no. 26; directive 2010/63/EU of the European Parliament and of the council) and were approved by the Ethics Committee of the IRCCS Regina Elena National Cancer Institute (N. 823/2016) and by the Italian Ministry of Health (N. 183/2017-PR)

To better detail the role of EMICORON in targeting *KRAS*-mutated CRC, experiments were performed in vitro on HCT-116 CRC cells, which were sensitive to EMICORON treatment in an in vivo model of dissemination metastases [50]. To this end, we showed that after treatment with EMICORON both *KRAS* mRNA and protein expression (Fig. 2a and b) were down-regulated in these cells, thus suggesting the direct targeting by this compound of G4 structures present in this gene. Interestingly, in our subsequent experiments in vivo performed in a panel of PDXs bearing *KRAS* mutations*,* we confirmed that this compound is very active against this tumor population (Fig. 3) for which no clinically effective therapy currently exists. In fact, we observed that EMICORON has a marked antitumoral activity against all the three different PDXs evaluated, as a reduced tumor mass was observed in treated mice compared to the untreated ones. Moreover, since most patients with advanced mutated *KRAS* mCRC and treated with systemic chemotherapy unfortunately showed disease progression, we investigated the ability of EMICORON to improve the efficacy of chemotherapy. In particular, EMICORON was added to the standard regimen FOLFIRI and the therapeutic efficacy of FOLFIRI alone was compared to the FOLFIRI plus EMICORON on CRC PDXs. As shown in Table I, after treatment with FOLFIRI one mouse exhibited tumor regression and three mice showed stabilization of the disease, while one mouse progressed. Interestingly, it is evident that the combination with EMICORON consistently improved the antitumor efficacy of FOLFIRI as regression in three mice treated and stabilization in the remaining two mice was observed. While the molecular mechanisms of EMICORON activity against *KRAS*-mutated tumors need to be fully elucidated, our data on HCT-116 cells showing the effect of EMICORON in down-regulating both *KRAS* mRNA and protein expression accompanied by the strong in vivo antitumor efficacy against a panel of *KRAS*-mutated CRC PDXs, suggest the potential of EMICORON for the clinical application of EMICORON in this mCRC clinical setting.

**Table 1:** EMICORON increase therapeutic efficacy of FOLFIRI regimen against CRC PDXs

Treatment^a^	Regression^b^	Stable disease^b^	Progression^b^
EMICORON	0/5	1/5	4/5
FOLFIRI	1/5	3/5	1/5
FOLFIRI plus EMICORON	3/5	2/5	0/5

^a^Tumor fragments derived from passage 1, were coated in Matrigel and implanted s.c. in mice (15–20 mm^3^/mouse) as described in the legend of fig. 2. When the tumor mass reached about 250 to 300 mm^3^, treatment started by giving mice FOLFIRI regimen consisting in Irinotecan (CPT-11) i.p. at 15 mg/kg for five consecutive days, followed by 5-Fluorouracil (5-FU) i.p. at 19 mg/kg plus Leucovorin at 20 mg/kg for five consecutive days. EMICORON was administered per os at 15 mg/kg for the next seven days. Five mice were included in each experimental group

^b^Regression or stable disease were considered when was observed the reduction or the maintenance, respectively, of the same tumor weight for at least two weeks after the starting of treatment

Moreover, this compound could be an attractive therapeutic also for *KRAS* wild type mCRC tumors, given that the majority of these patients acquired resistance to anti-EGFR monoclonal antibodies.

### Targeting KRAS by microRNAs

We devoted a separate chapter to the emerging role of microRNAs (miRNAs) in the regulation of KRAS for two main reasons: 1) the increasing interest in these molecules for their role in crucial biological aspect of cancer, including CRC; 2) the mechanism of action which includes both the binding to regulatory regions of *KRAS* or to genes involved in KRAS-driven pathways. MiRNAs are small (~ 18–25 nucleotide) non-coding RNAs which silence gene expression post-transcriptionally by binding to specific regions in the 3′UTR of mRNA [52]. Many studies have demonstrated that miRNAs play a key role in many crucial biological processes including cancer mechanisms such as cell proliferation, metastasis, apoptosis and angiogenesis [53].

The first set of evidence demonstrating the involvement of miRNA as tumor suppressor in human cancer was obtained in 2002 by Croce’s group from studies in B-cell chronic lymphocytic leukemia cells [54]. Nowadays, an increasing number of groups are identifying a number of miRNAs as potential biomarkers for human cancer diagnosis, prognosis and therapeutic targets. Aberrant expression of miRNAs has been reported for most types of cancers, including CRC [55], and in particular miRNAs regulate critical pathways involved in the CRC pathogenesis such as the p53, PI3K, RAS, MAPK, EMT transcription factors, and Wnt/β-catenin pathways [56].

The miRNA-mediated regulation of RAS protein was firstly identified in 2005 by Johnson’s group which described the well studied Let-7 family of miRNAs [57]. The human Let-7 family contains 13 members that regulate cell proliferation and differentiation by directly targeting various oncogenes and where the Let-7 expression is deregulated in many human cancers [58, 59]. In particular, Let-7b miRNA have been shown to repress KRAS expression and inhibit mutant KRAS-dependent cell growth in vitro and tumor growth in vivo in lung and pancreatic cancer cell models [60, 61].

The role of some miRNA can vary between the different cancer histotypes. In particular, miR-96 can act as either oncomirs or tumor suppressors depending on the cell context. For instance, its expression has increased in lung, prostate, bladder, colorectal and breast cancer [62]. By contrast, the expression of miR-96 is strongly down-regulated in pancreatic cancer. Yu et al. [63] demonstrated that miR-96 can affect cell proliferation, migration and apoptosis in pancreatic cancer cells by binding the 3’UTR region of KRASG12C mRNA, inhibit its protein expression and consequently decrease Akt signaling. Other miRNAs have been shown to behave like KRAS tumor suppressors, in particular miRNA-181a in oral squamous cell carcinoma [64], and finally miRNA-30b in CRC [65]. Moreover, it has been reported that reduced expression of miR-143 contributes to CRC development through the derepression of *KRAS* expression [66]. In fact, these authors observed an inverse correlation between KRAS protein and miR-143 and the treatment with miR-143 mimic reduced KRAS expression while miR-143 inhibitor increased KRAS protein level. Moreover, after treatment with miR-143 inhibitor CRC cells exhibited an increased cell proliferation, whereas miR-143 overexpression reduced the proliferation. These biological effects have been correlated to the direct recognition by miR-143 of the 3′-untranslated region of KRAS transcripts followed by the inhibition of constitutive phosphorylation of ERK1/2. More recently, by using a synthetic lethal strategy, Zhou et al. [67] identified miR-1298 and its targets, FAK and LAMB3, as potential suppressors of KRAS-dependent cell growth in both CRC and NSCLC cells. In fact, they observed that the miR-1298 mimic was lethal in all the *KRAS* mutant cells analyzed, both in vitro and in vivo. Moreover, miR-1298 functions by the binding and degradation of target FAK (focal adhesion kinase) and LAMB3 (laminin β3 subunit) which are transcriptionally regulated by mutant KRAS.

The role of miRNAs in the regulation of *KRAS* in the CRC context summarized here suggests that miRNAs could be a further potential strategy to explore effective targeting of KRAS-driven tumors.

### Conclusions

Even though it has been more than 30 years following the discovery of the role of *KRAS* in transforming cells and in driving cancer growth and development, there are no drugs in this current moment targeting the activating mutations of *KRAS* or selectively down-regulating KRAS mRNA and proteins, neither are there ones inhibiting downstream effector pathways in clinical trials. This represents a major problem in the treatment of three of the deadliest cancers, including pancreas, non-small cell lung and CRC, as *KRAS* mutations are associated with unfavourable prognosis in these tumor histotypes. In this review, we presented the most recent and promising advances in the development of new compounds able to target *KRAS*. We covered the many challenges emerging in this field of research, as most of these compounds are in the very early stages of preclinical experimentation or for other, mechanisms of action need to be elucidated. In summary, the road to discovering the first approved *KRAS* inhibitor is still long and winding, considering the genetic heterogeneity of *KRAS*-mutant cancers and the cell context. Nevertheless, these results represent fascinating efforts which could overcome the perception that *KRAS* is basically undruggable and that can help to develop effective drugs for targeting traditionally difficult pathways in the clinical setting.
